# Supplementary material for: Proteomics and Metabolomics Profiling of Pork Exudate Reveals Meat Spoilage during Storage
Source: Metabolites. 2022 Jun 21;12(7):570. doi: 10.3390/metabo12070570 (PMC9323900; doi:10.3390/metabo12070570)
Supplement: Supplementary file 1 [file metabolites-12-00570-s001.zip › Table S1.pdf]

Table S1. Identification of significantly differential metabolites in pork exudate during storage at four temperatures

Category	Name	VIP	Fold change				P value			
			-2 vs C	4 vs C	10 vs C	25 vs C	-2 vs C	4 vs C	10 vs C	25 vs C
Organic/Fatty acids	Stearolic acid	1.143	16.84	26.40	2.03	52.29	0.01	0.02	< 0.01	< 0.01
	9,10-Epoxystearic acid	1.004	87.21	136.24	2.59	71.95	< 0.01	0.02	< 0.01	< 0.01
	Juniperic acid	1.004	32.78	23.43	4.30	19.12	< 0.01	0.01	< 0.01	< 0.01
	4-Hydroxy-4-methylglutamic acid	1.161	0.38	0.32	0.28	0.08	< 0.01	< 0.01	< 0.01	< 0.01
	Ascorbic acid	1.206	0.16	0.16	0.63	0.15	< 0.01	< 0.01	< 0.01	< 0.01
	3-Deoxyarabinohexonic acid	1.181	0.17	0.36	0.26	0.33	0.01	< 0.01	< 0.01	< 0.01
	Isopropylmalic acid	1.007	84.46	41.37	0.26	11.88	< 0.01	0.05	< 0.01	< 0.01
	γ-Aminobutyric acid	1.139	0.09	0.08	0.03	0.03	< 0.01	< 0.01	< 0.01	< 0.01
Esters	Carnitine	1.087	0.19	0.13	0.07	0.10	0.02	0.01	0.01	0.01
	Tiglylcarnitine	1.116	0.21	0.10	0.22	0.22	< 0.01	< 0.01	< 0.01	< 0.01
Nucleotides/Nucleosides	5,6-Dihydrothymidine	1.186	3.19	3.09	3.60	2.22	< 0.01	< 0.01	< 0.01	< 0.01
	Dihydrothymine	1.129	2.84	2.54	2.68	2.08	< 0.01	< 0.01	< 0.01	< 0.01
	Inosine 5′-monophosphate	1.115	0.39	0.29	0.34	0.43	< 0.01	0.01	< 0.01	< 0.01
	Hypoxanthine	1.074	0.24	0.17	0.42	0.20	< 0.01	< 0.01	< 0.01	< 0.01
Peptides/Amino acids	Ala-Tyr	1.141	0.40	0.31	0.25	0.02	< 0.01	< 0.01	< 0.01	< 0.01
	Leu-Leu	1.160	0.10	0.08	0.04	0.02	< 0.01	< 0.01	< 0.01	< 0.01
	Leu-Val	1.138	0.04	0.03	0.07	0.02	< 0.01	< 0.01	< 0.01	< 0.01
	Val-Val	1.146	0.28	0.26	0.24	0.04	< 0.01	< 0.01	< 0.01	< 0.01
	Arginine	1.129	0.05	0.04	0.03	0.13	0.01	0.01	< 0.01	0.01
Lipids	PC(34:4)	1.038	4.51	11.74	0.41	0.40	< 0.01	0.03	< 0.01	< 0.01
	PC(36:3)	1.467	11.54	11.55	13.68	8.31	0.01	< 0.01	0.01	0.02
Carbohydrates	Fructose 6-phosphate	1.150	0.07	0.08	0.05	0.15	< 0.01	< 0.01	< 0.01	< 0.01
	Glucose 6-phosphate	1.125	0.08	0.08	0.05	0.21	< 0.01	< 0.01	< 0.01	< 0.01
Alcohols	Prosopinine	1.087	10.66	9.77	2.66	98.96	< 0.01	0.02	< 0.01	0.02
	Estrane-3,17-diol	1.126	4.03	6.33	0.44	36.96	< 0.01	0.04	0.03	0.01
Ketone	6-Hydroxypseudooxynicotine	1.238	57.60	60.18	4.40	126.45	< 0.01	0.03	< 0.01	< 0.01
	Farnesylacetone	1.155	16.98	26.82	2.08	52.86	0.01	0.02	< 0.01	< 0.01
Others	Hexose	1.109	0.08	0.12	0.26	0.12	< 0.01	< 0.01	< 0.01	< 0.01
	Stearoyl Ethanolamide	1.076	2.28	3.56	5.03	5.87	0.01	< 0.01	< 0.01	< 0.01
	Dibenzylamine	1.135	0.39	0.29	2.32	0.39	< 0.01	0.01	< 0.01	< 0.01
