# Supplementary figures and images for: Proteomics and Metabolomics Profiling of Pork Exudate Reveals Meat Spoilage during Storage
Source: Metabolites. 2022 Jun 21;12(7):570. doi: 10.3390/metabo12070570 (PMC9323900; doi:10.3390/metabo12070570)

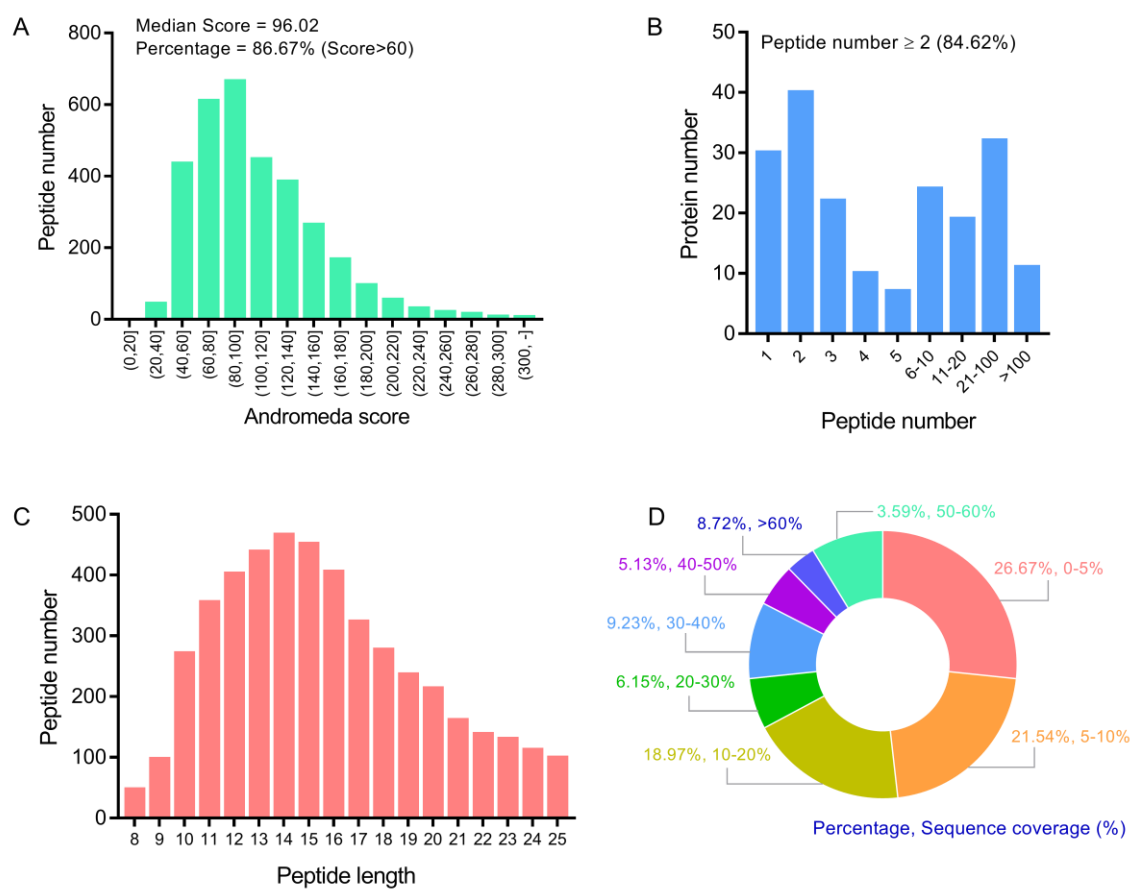

Figure S1.

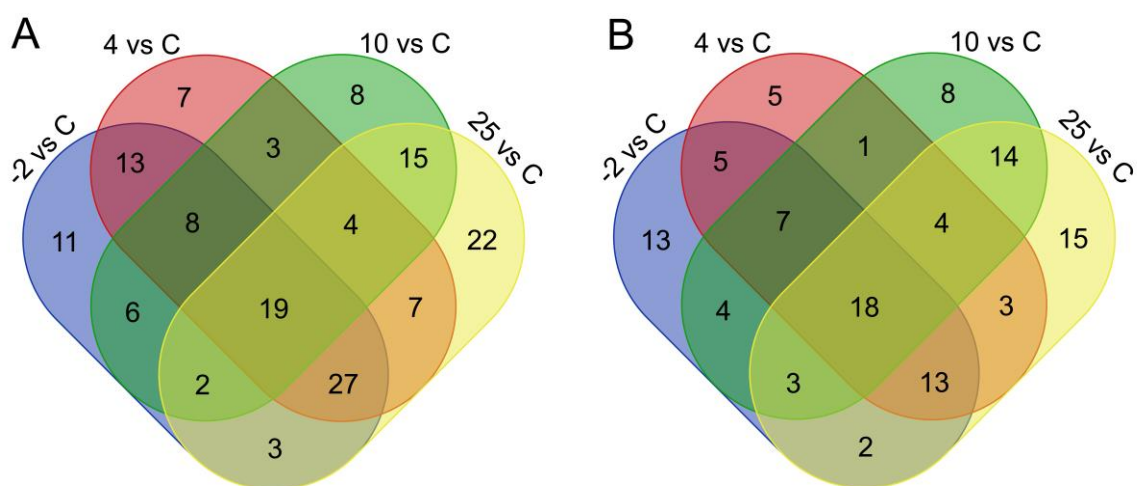

Figure S2.

Supplement: Supplementary file 1 [file metabolites-12-00570-s001.zip › metabolites-1754759-Supplementary Figures.pdf]
